# Microlaparoscopy in Sex Reassignment Surgery

**DOI:** 10.1100/tsw.2004.53

**Published:** 2004-06-07

**Authors:** S. De Stefani, C. Trombetta, M. Raber, G. Savaco, U. Moro, E. Belgrano

**Affiliations:** Intituto di Clinica Urologica Universitá-spedaleCattinara, Trieste-Italy, Italy

## Abstract

Sex reassignment (male to female surgery) is a standard operation which is aimed at constructing female genitalia and obtaining a cosmetic and functional result that is similar to that of a normal female subject. The ideal surgical procedure has not yet been described, but the various techniques which have been proposed in the literature are similar. The most cumbersome maneuver of the procedure is that of creating a neovaginal cavity inside the perineum. This step is generally carried out by means of blunt dissection between the rectal wall and the prostate, but most of the surgery is blindly performed without visual control. In these conditions, the risk of rectal injury is high, and may lead to severe intraoperative complications. Microlaparoscopy allows for a direct observation of the perineal dissection from inside the peritoneal cavity, thus avoiding risk of rectal injury. The technique is simple to perform, is non-invasive, and only 15 minutes are added to the operation.

## INTRODUCTION

Sex reassignment male to female surgery is a standard operation, the technical details of which have been fully described by many authors in recent reports. The operation may be divided into six main surgical steps which include castration, penectomy, vulvoplasty and dissection of a perineal space for the neovagina. This last maneuver is sometimes troublesome and requires surgical refinement. In fact, rectal lesions which may lead to temporary enterostomy, have been described. The vaginal cavity is commonly created by blunt dissection which is performed between the bulbocavernous muscle, Denonvilliers' fascia ventrally and the levator ani muscle and the rectum dorsally and laterally. Dissection is carried distally for about 12 cm until the peritoneum is reached at the rectovesical region. At this site, the risk of peritoneal perforation and rectal laceration is high because the surgical field is located deep in the pelvis and the maneuver is completely "blind". Microlaparoscopy is very useful in these cases because the dissection of the rectovesical space may be guided directly, thus avoiding accidental injury of the surrounding structures. The procedure is quite simple and is noninvasive.

## CASE REPORT

A 23 year-old patient was diagnosed as a transsexual and scheduled for sex reassignment surgery. A circumferential incision was carried out in the prepuce, 1 cm below the coronal sulcus, the penile skin was then mobilized and pulled down to the base of the shaft. The urethra, with its corpus spongiosum, was freed from the corpora cavernosa up to the glans. The neurovascular bundle of the penis was detached from the dorsal surface of the corpora by microsurgical technique and the glans was separated from the apex of the carvernous bodies. A midline incision was made in the perineum, beginning proximally about 1 cm above the base of the penis and extended toward the anus. The incision was extended proximally to an inverted Y with one angle on either side of the anus. Through this incision the corpora cavernosa were exposed up to the crura, which were severed in succession. The two corporal stumps were sutured together over the symphysis pubis. The glans was dissected and molded to form a clitoris. Great care was given to save its vascular nourishment. Bilateral orchiectomy was performed. The distal end of the penile skin was inverted and sutured to form a pouch. After cutting the ventral part of the central tendon, a vaginal cavity was created in the pelvis. The prostato-rectal ligaments were severed and the dissection was continued inward until the peritoneum was reached at the rectovesical region, dorsal to the prostate, outside Denonvilliers' fascia. This step of the operation was monitored by the use of microlaparoscopy. An umbilical incision was made and the Veress needle was inserted. Insufflation with $CO_2$ was accomplished in the usual fashion, setting the intra-abdominal pressure at a level no greater than 8-10 mm and a maximum volume of gas of 3 L. Once the intra-abdominal pressure reached 8 mm, insufflation was stopped and the 1.75 mm microendoscope inserted. The rectovesical region was directly visualized and the dissection carried out through the perineal approach. Two stitches were passed between the distal end of the cutaneous pouch and the bottom of the perineal cavity. The passage of the needles could be observed by the microendoscope. At the end of the procedure, the microendoscope was removed and the pneumoperitoneum was completely deflated via the Veress needle.

The urethra was severed and a urethrostomy performed through a slit in the skin lining the neovagina, below the symphysis pubis. The operation ended with the formation of labia obtained with the scrotal skin and the construction of a pseudoclitoris.

## CONCLUSION

Sex reassignment male to female surgery is a reconstructive operation aimed at obtaining a good cosmetic appearance. Surgical techniques are well codified and relatively simple to perform. Microsurgery is useful for performing the most delicate procedures such as neurovascular bundle dissection, glans conversion into clitoris and urethral dissection and reanastomosis. Postoperative complications are generally not severe and include the possible stenosis of the new urethral meatus, the necrosis of the glans and urethral fistulas. These may be avoided with experience and the use of atraumatic techniques during the operation. Stenosis of the neovagina is generally due to improper postoperative dilation by the patient. Rectal injury during the perineal dissection is a severe intraoperative complication. The use of microlaparoscopy avoids the risk of this complication and allows some complementary maneuvers such as the fixation of the apex of the neovagina to the bottom of the perineal cavity by means of a few sutures. This procedure allows the hitching of the neovagina deeply in the perineum and a better functional and cosmetic result.

The surgical reconstruction of a well functioning vagina is important but not the only aim of this operation. Psychological evaluation and support are required before surgery in order to select the patients who can benefit from the operation. Aid in obtaining a complete social adjustment subsequently is also important.

In conclusion, the microendoscope allows for a minimally invasive approach for sex reassignment surgery. Microlaparoscopy provides direct observation of the perineal dissection from inside the peritoneal cavity, thus avoiding the risk of rectal injury. The technique is simple to perform, is noninvasive and only 15 minutes are added to the operation.
